# Food Security Sensing System Using a Waveguide Antenna Microwave Imaging through an Example of an Egg

**DOI:** 10.3390/s20030699

**Published:** 2020-01-27

**Authors:** Tzu-Chun Tai, Hung-Wei Wu, Cheng-Yuan Hung, Yeong-Her Wang

**Affiliations:** 1 Department of Photonics, National Cheng Kung University, Tainan 701, Taiwan; L78051041@gs.ncku.edu.tw; 2 Department of Information and Communication, Kun Shan University, Tainan 710, Taiwan; 3 Opto-Electronics Technology Section Energy and Agile System Department, Metal Industries Research and Development Centre, Kaohsiung 821, Taiwan; goliro.goliro@gmail.com

**Keywords:** sensing, antenna, microwave imaging, egg, food security

## Abstract

In this paper, we present a form of food security sensing using a waveguide antenna microwave imaging system through an example of an egg. A waveguide antenna system with a frequency range of 7–13 GHz and a maximum gain of 17.37 dBi was proposed. The maximum scanning area of the waveguide antenna microwave imaging sensing system is 30 × 30 cm^2^. In order to study the resolution and sensitivity of the waveguide antenna microwave imaging sensing system, the circular and triangular high-*k* materials (with the same thickness but with different dielectric constants of the materials) were used as the testing sample for observing the microwave images. By using the proposed waveguide antenna microwave imaging sensing system, the high-*k* materials with different dielectric constants and shapes could be easily sensed. Therefore, the waveguide antenna microwave imaging sensing system could be potentially used for applications in rapid, non-destructive food security sensing. Regarding the example of an egg, the proposed waveguide antenna microwave imaging sensing system could effectively identify the health status of many eggs very quickly. The proposed waveguide antenna microwave imaging sensing system provides a simple, non-destructive, effective, and rapid method for food security applications.

## 1. Introduction

Microwave imaging technology is defined as “roughly and quickly [seeing]” (means for initial rapid screening) the hidden objects in an object’s internal structure by means of electromagnetic fields at microwave frequencies (300 MHz–30 GHz). Microwave imaging technologies and systems have been widely studied and discussed in terms of developing agricultural, safety, industrial, and medicinal applications. Microwave images are maps of the electrical property distributions in dielectric samples [1,2].

In the microwave imaging system, the antenna is a crucial component. Moreover, the use of an antenna is a critical aspect and influences the resolution and sensitivity of the output image. In Ahadi et al. [3], an ultra-wideband (UWB) antenna with UWB characteristics in a medium emulating breast tissue was presented. In Ojaroudi et al. [4], a UWB printed monopole antenna was proposed for use in a microwave imaging system. The antenna has an optimal radiation pattern, even at higher frequencies, and its radiation efficiency is greater than 86%. In Capobianco et al. [5], a planar antenna was proposed for use in an imaging antenna array that was operated in a broad frequency range of 3–18 GHz. In Kang et al. [6], a ground-folded slot antenna was proposed to enhance the gain and reduce the backward radiation of a microwave image. In Yurduseven et al. [7], a frequency-diverse aperture was proposed for use in a microwave imaging system based on a planar cavity. In the experiment, a number of metal targets were used, including a 2-cm resolution target, a gun phantom, and two L-shaped phantoms. In References [8,9,10], a circular array of antennas with UWB pulses and backscattered signals for early-stage breast cancer detection, a multi-static ultra-wideband antenna array configuration, and a UWB aperture horn antenna raster scanning technique were proposed. These studies have greatly inspired us to consider improving the performance of microwave image systems. However, it is noted that the challenge to improve the efficiency of the antenna and to optimize the high-resolution algorithm in the microwave image system is crucial. 

Currently, the Haugh unit (HU) is a commonly used method for classifying the quality of eggs [11]. According to the conventional HU detection method, the egg must be broken to measure the height of the yolk and albumen; if the eggs are spoiled, moisture in the eggs is lost and the HU can calculate the freshness of eggs [12]. Dielectric properties as a function of temperature and frequency have been reported for different agricultural commodities, such as grains, seeds, fruits, vegetables, albumen solutions, and thermally denatured albumen gels [13,14]. These studies have reported the possibility of destructively predicting the electrical characteristics of agri-food products, such as eggs. In Ragni et al. [13], the dielectric properties of fresh eggs during storage were investigated. The dielectric properties were determined using an open-ended coaxial probe on the egg yolks after 1–15 days of storage at room temperature. In Guo et al. [14], the dielectric properties of egg albumen and yolk were apparently distinguished over the frequency range of 10–1800 MHz. However, the conventional HU method has some disadvantages, namely its destructive detection and low accuracy. Moreover, as the storage time of an egg increases, the internal albumen is evaporated into the air through the eggshell, resulting in a decrease in internal albumen capacity. There is no effective method to detect and observe the hidden safety of an egg.

In this study, we proposed a new microwave imaging sensing system and its application toward food security for the first time. A waveguide horn antenna with a metal waveguide as the transmitting waveguide antenna with a scanning frequency range of 7–13 GHz and maximum gain of 17.37 dBi was proposed. The transmitting waveguide antenna could effectively improve the resolution of the imaging samples and the ability to observe the content capacity of the samples. The scanning frequency and dielectric properties of the testing sample were the two aspects of the microwave imaging sensing system that were considered. Additionally, this was the first time a microwave imaging algorithm based on the measured S-parameters for further optimizing and correcting the microwave image deformation was proposed. We first used circular and triangular high-*k* dielectric materials (with the same thickness but with different dielectric constants) as the testing sample for observing the microwave images of the materials with different dielectric properties, thicknesses, and shape. After confirming the image resolution and efficiency of the waveguide antenna microwave imaging sensing system, a fresh egg and an egg with less albumen (not fresh) were sensed using the microwave imaging sensing system. The sensing results revealed the optimal capability of the system in evaluating the health of eggs. The system can be potentially used in the applications of rapid, non-destructive food security sensing. The proposed system provides a simple, non-destructive, effective, and rapid method for food security applications.

## 2. Construction and Analysis of the Waveguide Antenna Microwave Imaging Sensing System

**Figure 1:**
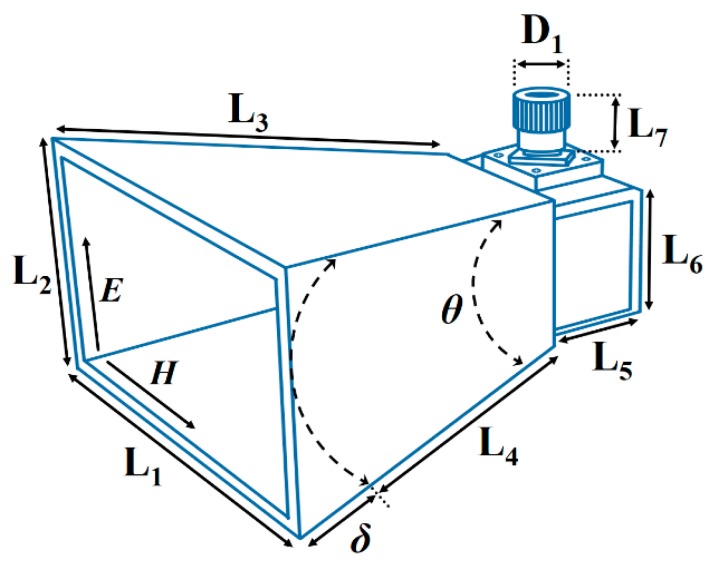
Configuration of the transmitting waveguide antenna. L_1_ = 95, L_2_ = 78, L_3_ = 76, L_5_ = 33, L_6_ = 15, L_7_ = 10, D_1_ = 3.4 (all are in mm).

The configuration of the transmitting waveguide antenna is displayed in Figure 1. The waveguide antenna was used as the transmitting waveguide antenna (TWA). The transmitting waveguide antenna consisted of horn-shaped flared metal, which was used to direct radiation waves in the electromagnetic (EM) beam. To design the transmitting waveguide antenna in this study, the critical configuration parameters of the transmitting waveguide antenna were the opening angle *θ*, the aperture of the mouth L_2_, the length L_4_, and the path length difference *δ*. From the geometric model, we found that L_4_ = L_2_^2^/8*δ* and *θ* = 2cos^−1^(L_4_/L_4_ + *δ*). In the *E* plane of the transmitting waveguide antenna, *δ* was usually about 0.25λ, while in the *H* plane of the transmitting waveguide antenna, *δ* was about 0.4λ. The radiation pattern and gain of the transmitting waveguide antenna could be decided through adjusting the length L_3_ and the opening angle *θ*. The characteristics of the proposed transmitting waveguide antenna were then designed and analyzed using an HFSS Simulator (ver. 14.0, Ansoft, Palo Alto, CA, USA). The advantages of the transmitting waveguide antenna were as follows: moderate directivity, low standing wave ratio, wide bandwidth, and simple construction and adjustment. The high directivity horn antenna could reduce the backward radiation to provide good stability in the radiation region and scanning plane. The transmitting waveguide antenna could effectively enhance the resolution of the microwave images. The half-power beam width (HPBW) of the transmitting waveguide antenna was approximately 12.5° in the X–Z and Y–Z planes at each scanning frequency. Based on the design of the HPBW, the optimal resolution region was predicted to be approximately 9.6 × 9.6 cm^2^. The S-parameter (−10log|S_11_|) of the transmitting waveguide antenna was approximately 10 dB in the frequency range of 7–13 GHz. The frequency was selected by considering safety applications among those envisioned by Federal Communications Commission (FCC) authorization of ultra-wideband technology [15]. An imaging sensing system may be used for a variety of health applications to “see” an object’s internal structure. The dimensions of the transmitting waveguide antenna were 95 × 78 × 109 mm^3^. 

**Figure 2:**
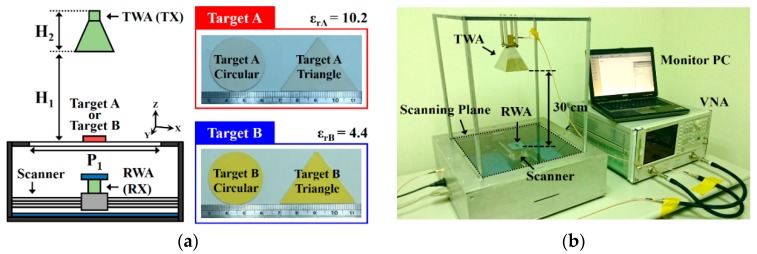
Configuration of the waveguide antenna microwave imaging sensing system. (**a**) The material thickness of target A and target B were 0.8 mm and 2.5 mm, respectively, and (**b**) photograph of the waveguide antenna microwave imaging sensing system. TWA(TX): transmitting waveguide antenna, RWA(RX): receiving waveguide antenna, and VNA: vector network analyzer. Target A was a ceramic–polytetrafluoroethylene composite and target B was a woven fiberglass cloth and epoxy resin binder. P_1_ = 300 mm, H_1_ = 300 mm, and H_2_ = 109 mm. The distance between the transmitting waveguide antenna and the scanning plane of the system was fixed at 30 cm and the scanning area was around 30 × 30 cm^2^. The sensing target was placed in the center of the scanning plane.

The configuration of the waveguide antenna microwave imaging sensing system is displayed in Figure 2. The transmitting waveguide antenna was installed on top of the system and the receiving waveguide antenna was set on the bottom of the system. The configuration of the waveguide antenna microwave imaging platform was divided into four steps. First, to define the scanning area of the platform, the scanning area of 30 × 30 cm^2^ was demonstrated in this study. Second, the effective distance between the transmitting waveguide antenna and the scanning plane for achieving the optimal resolution of the microwave image was determined. Third, the transmitting waveguide antenna radiation angle for covering the entire scanning plane was designed. Fourth, the scope and collection points of the S-shape scanning path of the receiving waveguide antenna to ensure the microwave images were not distorted was determined. To study the resolution and sensitivity of the system, we first used circular and triangular high-*k* dielectric materials as the testing sample for observing the microwave images of the materials with different dielectric properties. Target A was a ceramic–polytetrafluoroethylene composite with a dielectric constant (*ε*_rA_) of 10.2. Target B was a woven fiberglass cloth and epoxy resin binder with a dielectric constant (*ε*_rB_) of 4.4. Targets A and B had thicknesses (t_B_) of 0.8 and 2.5 mm, respectively. The scanning frequency of the waveguide antenna microwave imaging sensing system was set to 8.4, 9.2, and 10.4 GHz. The HP 8510C vector network analyzer (Keysight Technologies Inc., Santa Rosa, CA, USA) was connected to a microwave imaging platform. When a high-gain transmitting waveguide antenna passed the signal through the testing sample to receiving end, the receiving waveguide antenna (RWA) needed to match the transmitting waveguide antenna to decrease the radiation interference of both antennas. The receiving waveguide antenna used a rectangular waveguide configuration. The contribution of the receiving waveguide antenna was kept stable to receive the signal from the transmitting waveguide antenna. The receiving waveguide antenna with a scanning frequency range of 7.2–14.3 GHz was used. In addition, the receiving waveguide antenna scanned along the S-shape path for the scanning area (30 × 30 cm^2^) within 600 s. A total of 2601 frequency sampling points were extracted as the sum of 51 × 51 scanning points in the X-Y axis.

**Figure 3:**
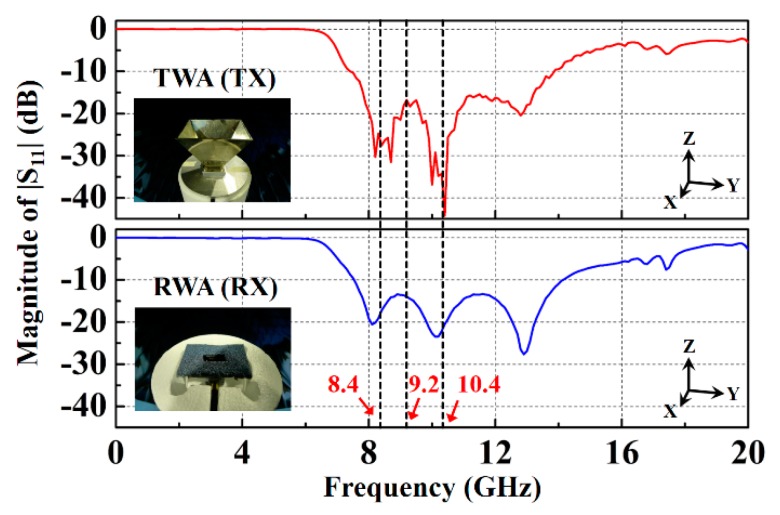
Measured magnitude |S_11_| of the transmitting waveguide antenna and the receiving waveguide antenna.

The measured magnitudes of |S_11_| of the transmitting waveguide antenna and receiving waveguide antenna are presented in Figure 3. The transmitting waveguide antenna was designed to have an |S_11_| of more than 10 dB in the frequency range of 7–13 GHz. The receiving waveguide antenna was designed to have an |S_11_| of more than 10 dB in the frequency range of 7.6–14.1 GHz. The scanning frequency of the system was set to 8.4, 9.2, and 10.4 GHz. The magnitudes of |S_11_| of 27.6 and 17.26 dB, 16.7 and 13.96 dB, and 44.28 and 20.52 dB corresponded to the transmitting waveguide antenna and receiving waveguide antenna at scanning frequencies of 8.4, 9.2, and 10.4 GHz, respectively. The scanning frequency of the waveguide antenna microwave imaging sensing system was related to the quality of microwave image resolution. Through studying the dielectric properties of the high-k materials, it was found that the scanning frequency of 10.4 GHz was useful for enhancing the microwave image resolution.

**Figure 4:**
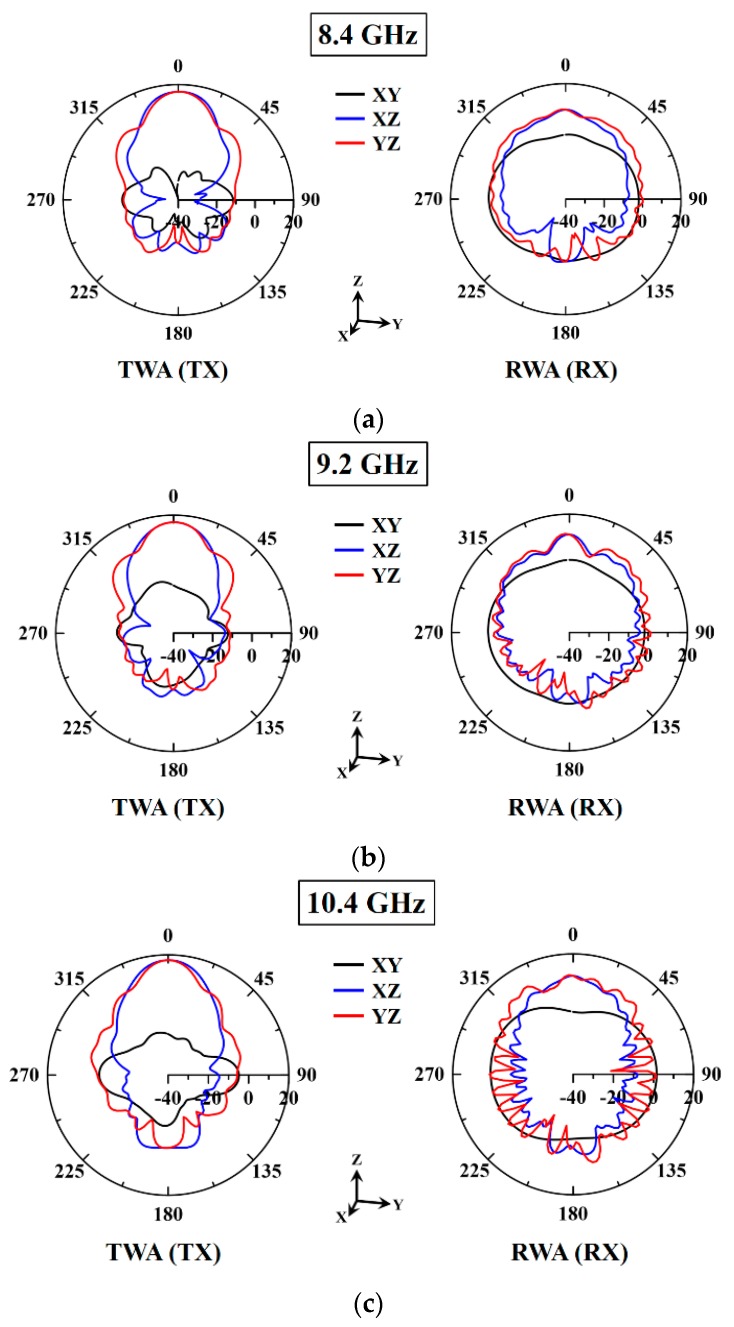
Measured two-dimensional radiation pattern on the X–Y, X–Z, and Y–Z planes of the transmitting and receiving waveguide antennas at scanning frequencies of (**a**) 8.4 GHz, (**b**) 9.2 GHz, and (**c**) 10.4 GHz.

Figure 4 presents the measured two-dimensional radiation pattern in the x–y, x–z, and y–z planes of the transmitting and receiving waveguide antennas at scanning frequencies of 8.4, 9.2, and 10.4 GHz. The gain and radiation pattern of the transmitting and receiving waveguide antennas were measured using the SATIMO antenna measurement equipment (StarLab, Microwave Vision Group, FR). The measured gains of the transmitting waveguide antenna were 16.28, 16.4, and 17.37 dBi at the scanning frequencies of 8.4, 9.2, and 10.4 GHz, respectively. The radiation pattern of the transmitting waveguide antenna presented the maximum measured gain of 17.37 dBi at 10.4 GHz. The measured gains for the receiving waveguide antenna were 6.5, 9.73, and 9.11 dBi at the scanning frequencies of 8.4, 9.2, and 10.4 GHz, respectively. The S-parameter (|S_11_|) of the antenna at 9.2 GHz was not optimal; however, this did not affect the microwave imaging resolution. The image resolution was highly related to the measured gain and scanning frequency of the transmitting waveguide antenna.

**Figure 5:**
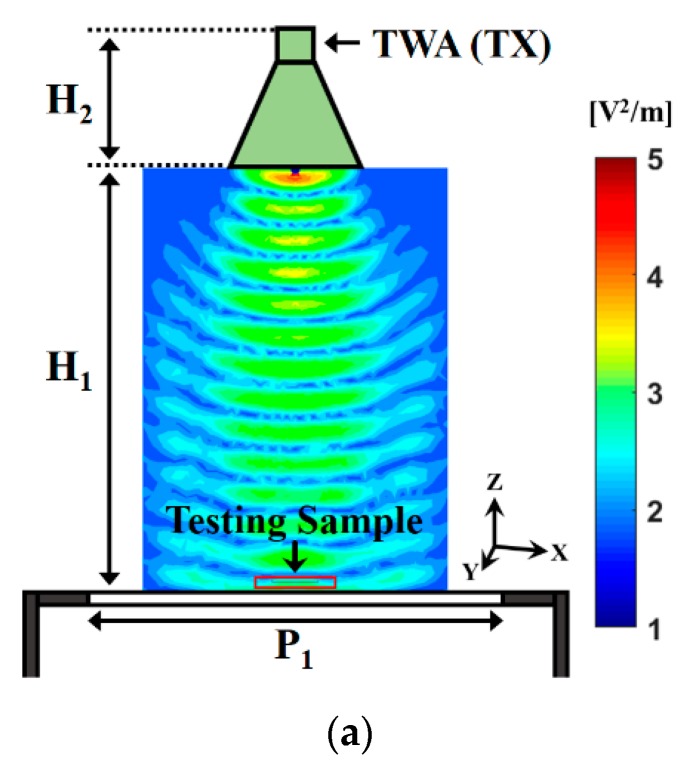

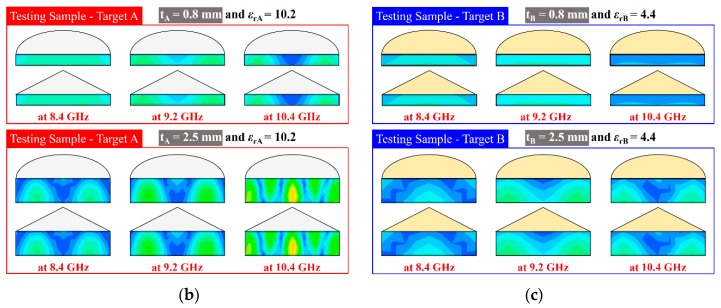
Simulated cross-sectional electric field distribution of the (**a**) waveguide antenna microwave imaging sensing system, (**b**) inside target A, and (**c**) inside target B. P_1_ = 300 mm, H_1_ = 300 mm, and H_2_ = 109 mm.

In order to enhance the performance of the proposed microwave image system, the use of 3D EM simulation Ansoft HFSS was considered first. The ceramic-based testing samples with different dielectric constants, shapes, and thicknesses (defined as target A and target B) were used to verify the capability of the proposed microwave imaging system. Figure 5 presents the simulated cross-sectional electric field distribution of the waveguide antenna microwave imaging sensing system. The comprehensive numerical evaluation of the performance of this system was conducted using the finite-difference frequency-domain method. Target A (*ε*_rA_ = 10.2) and target B (*ε*_rB_ = 4.4) were placed on top of the scanning plane of the system. The electrical field distribution of the target was affected by different dielectric properties. Target A had a stronger electric field than target B. Therefore, the electric field distributions of the target with a higher dielectric constant, which was obtained using the system, were highly affected. The algorithm for extracting the imaging data could be obtained using the measured complex propagation constant as follows: γ(*f*) = α(*f*) + jβ(*f*). This equation was derived from the ABCD transmission matrix transformed from the four measured scattering parameters (S_11_, S_12_, S_21_, and S_22_) of the transmission model, where α is the attenuation loss and β is related to the phase and dielectric constant of the eigenvalues. By using S-parameters and a field-mapping algorithm to compare and correct the image edge deformations, the proposed waveguide antenna microwave imaging sensing system could effectively improve the quality of the microwave imaging. To investigate the changes in the dielectric constant of the testing sample that was extracted from the phase of the transmission model [16,17,18], the following equations were used:

(1) $${\left[\begin{array}{cc} S11 & S12\\ S21 & S22 \end{array}\right]}_{\mathrm{DUT}}={\left[\begin{array}{cc} S11 & S12\\ S21 & S22 \end{array}\right]}_{\mathrm{total}}-{\left[\begin{array}{cc} S11 & S12\\ S21 & S22 \end{array}\right]}_{\mathrm{noise}}$$

(2) $${\left[\begin{array}{cc} S11 & S12\\ S21 & S22 \end{array}\right]}_{\mathrm{DUT}}\Leftrightarrow {\left[\begin{array}{cc} A=\frac{\left(1+S_{11}\right)\left(1-S_{22}\right)+S_{12}S_{21}}{2S_{21}} & B=Z_{0}\cdot \frac{\left(1+S_{11}\right)\left(1+S_{22}\right)-S_{12}S_{21}}{2S_{21}}\\ C=\frac{1}{Z_{0}}\cdot \frac{\left(1-S_{11}\right)\left(1-S_{22}\right)-S_{12}S_{21}}{2S_{21}} & D=\frac{\left(1-S_{11}\right)\left(1+S_{22}\right)+S_{12}S_{21}}{2S_{21}} \end{array}\right]}_{\mathrm{DUT}}$$

where [S]_DUT_, [S]_total_, and [S]_noise_ indicate the measured parameter values of the waveguide antenna imaging sensing system. The device under test (DUT) is the testing sample used for the imaging sensing system. Total and noise S-matrices are related to the presence and the absence of the testing sample on the scanning plane, respectively. The *β* of the testing sample can be found using Equation (3):

(3) β(f)=Im{ln[2S21(1-S221)(1-S222)+S12S21(2-2S11S22)+2S21(S12S21)2]}DUT =πf[2(εr+1)]1/2c(rad/m)

where *ε*_r_ indicates the testing sample and *c* indicates the speed of light.

## 3. Results

### 3.1. Feasibility Verification of the Waveguide Antenna Microwave Imaging Sensing System

**Figure 6:**
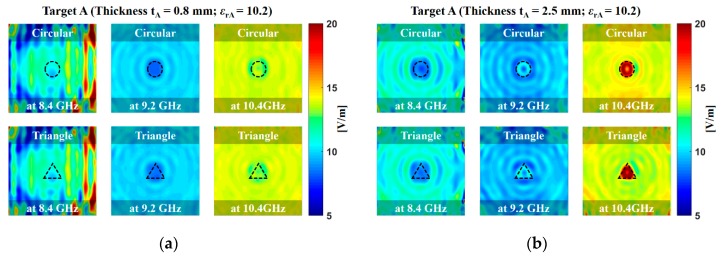
Measured microwave image of the target A with the thicknesses of (**a**) 0.8 mm and (**b**) 2.5 mm at scanning frequencies of 8.4 GHz, 9.2 GHz, and 10.4 GHz. Target A was a ceramic–polytetrafluoroethylene composite with a dielectric constant (*ε*_rA_) of 10.2. A total of 2601 frequency sampling points were extracted by using HP 8510C vector network analyzers (VNAs).

**Figure 7:**
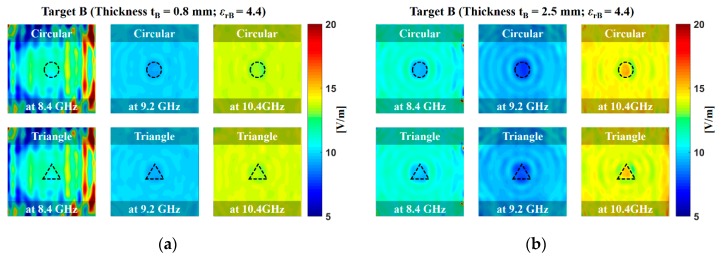
Measured microwave image of target B with the thicknesses of (**a**) 0.8 mm and (**b**) 2.5 mm at scanning frequencies of 8.4 GHz, 9.2 GHz, and 10.4 GHz. Target B was a woven fiberglass cloth and epoxy resin binder with the dielectric constant (*ε*_rB_) of 4.4. A total of 2601 frequency sampling points were extracted by using HP 8510C VNAs.

Figure 6 and Figure 7 display the obtained microwave image of targets A and B (the photograph of the testing sample is found in Figure 2) at scanning frequencies of 8.4, 9.2, and 10.4 GHz. The microwave image was obtained by reconstructing the received signals in the space-frequency domain by using a frequency-domain back-projection algorithm [19]. The reconstructed target image appeared at the center of the x- and y-axes and at approximately 30 cm on the z-axis, thus representing the exact position of targets A and B. The microwave imaging procedure was as follows. First, an acquisition step was performed for the transmitting and receiving waveguide antennas. Second, the targets were placed on the center of the scanning plane of the system. The procedure was repeated for multiple locations of the movable support. The background data were subtracted from the data obtained in the presence of the target, and by using a field-mapping algorithm, the electromagnetic fields from a surface to another were transformed in a sense. This algorithm used the form E(r) = T(r, rs)[Et(rs)], where r is any point vector in space, rs is a point vector on the data surface, E(r) the electric field at any r, Et(rs) is the tangential electric field at rs, while T(r, rs) transforms the fields from surface S(rs) to another S(r) [19]. The image resolution of the target shape was increased by increasing the scanning frequency from 8.4 to 10.4 GHz. The image resolution of the target with a thickness of 2.5 mm was superior to that of the target with a thickness of 0.8 mm. For a dielectric material, an applied electric field E causes the polarization of the atoms or molecules of the testing sample to create electric dipole moments. The complex dielectric constant of the testing sample can be an imaginary part as follows [20]:

(4) $$\varepsilon =\varepsilon^{\prime }-j\varepsilon^{\prime \prime }=\varepsilon_{0}(1+x_{e})$$

where *x_e_* is the electric susceptibility, *ε*_0_ is dielectric constant in the vacuum space, *ε′* is the real part of the dielectric constant and *ε*″ is a loss in the testing sample due to damping of the vibrating dipole moments. The dielectric loss of a testing sample can be considered an equivalent conductance in the lumped circuit model of the transmission line theory. In any testing sample with conductivity *σ*, the conduction current density will exist and is related to *H* as follows [20]:

(5) $$\nabla \times \bar{H}=j\omega \varepsilon^{\prime }\bar{E}+(\omega \varepsilon^{\prime \prime }+\sigma )\bar{E}=j\omega (\varepsilon^{\prime }-j\varepsilon^{\prime \prime }-j\frac{\sigma }{\omega })\bar{E}$$

where the term of *ωε*″+ *σ* can be considered the total conductivity. It can be comprehended that the electromagnetic wave is significantly affected by the conductivity in the testing sample. The high-conductivity materials have higher electromagnetic wave attenuation and effects than low-conductivity materials. Therefore, it is necessary to use the low-conductivity insulating materials as targets A and B in this study.

### 3.2. Food Security Sensing through an Example of an Egg

**Figure 8:**
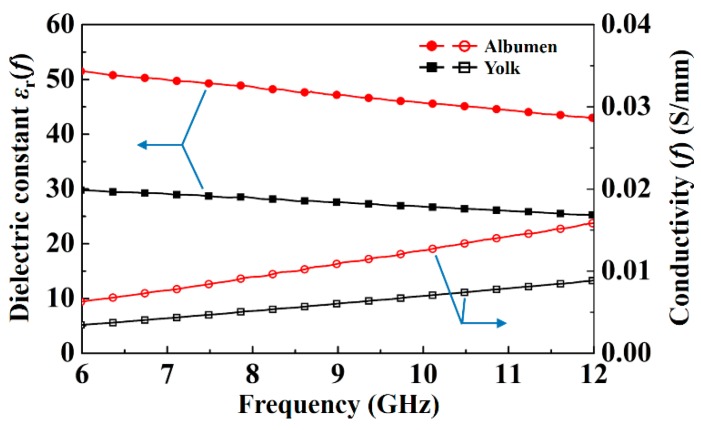
Measured frequency-dependent dielectric constant ε_r_ and conductivity of yolk and albumen at X-band.

We successfully sensed the circular and triangular high-*k* dielectric materials for observing the microwave images of the materials with different dielectric properties. After studying the image resolution and efficiency of the system, a fresh egg and an egg with less albumen (not fresh) were sensed using the proposed system. In general, there are no effective methods for rapidly and non-destructively sensing the health status of a large number of eggs. Microwave imaging technology can be an optimal solution to this problem and can be used for food security sensing. Figure 8 displays the measured frequency-dependent dielectric constant *ε*_r_ and conductivity of yolk and albumen for the X-band by using an open-ended coaxial probe method [21]. As the scanning frequency was increased, the dielectric constant of the albumen and yolk decreased, and the conductivity of the albumen and yolk increased. It was found that the difference in dielectric constant between the albumen and yolk needed to be over 20 in the X-band to have clear microwave imaging quality. It is noted that if the difference in dielectric constant between the albumen and yolk was close to 5, the quality of the microwave imaging declined. The detailed dielectric properties and conductivity of the measured albumen and yolk is displayed in Table 1.

**Figure 9:**
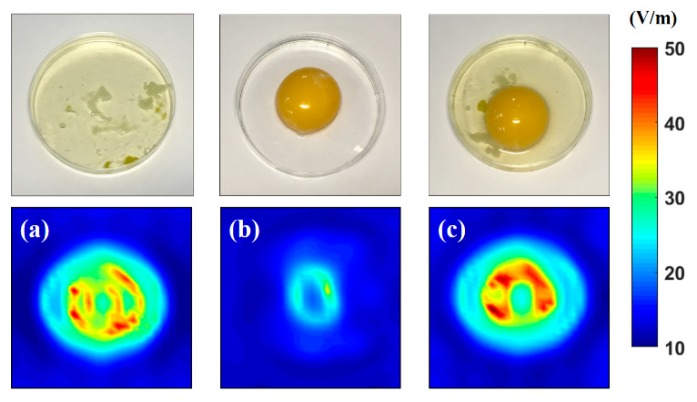
Microwave images of (**a**) only albumen, (**b**) only yolk, and (**c**) the combination of albumen and yolk (all of the microwave images were scanned at 10.4 GHz).

Figure 9 displays the microwave images of the albumen, the yolk, and a combination of the albumen and yolk. In Figure 9a, the microwave image of the albumen displayed a double-ring-like high electric field (color: yellow to red). The double-ring-like high electrical field indicated that the thick albumen (the dielectric constants of the components were in the following order: thick albumen > thin albumen > yolk) [13]. The thick albumen was clearly distributed in the high amount of thin albumen. As presented in Figure 9b, the microwave image of the yolk presented a circle-like shape. The dielectric constant of the yolk was lower than those of the thin albumen and thick albumen. Therefore, a weak electrical field was observed in the image of the combination of albumen and yolk. In the figure, the thin albumen, thick albumen, and yolk could be easily distinguished. As presented in the microwave image shown in Figure 9c, the yolk was evidently surrounded by the thick albumen layer. Therefore, the proposed system can be used to effectively identify the health of an egg. 

**Figure 10:**
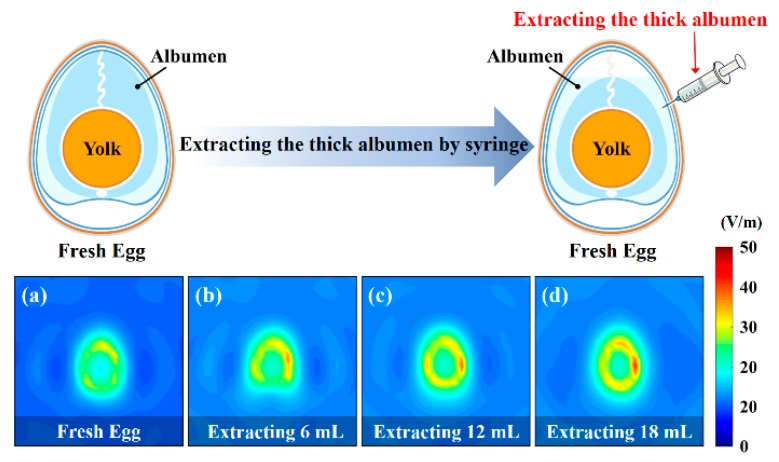
The health sensing of (**a**) a fresh egg, (**b**) a fresh egg after extracting 6 mL thick albumen, (**c**) a fresh egg after extracting 12 mL thick albumen, and (**d**) a fresh egg after extracting 18 mL thick albumen by using the waveguide antenna microwave imaging sensing system (all of the microwave images were scanned at 10.4 GHz).

Figure 10 displays the health-sensing results obtained for a fresh egg and of fresh eggs from which 6-, 12-, and 18-mL of thick albumen were extracted. To determine the sensitivity of the proposed system, we used a fresh egg as the control group and compared this egg with the eggs from which 6-, 12-, and 18-mL of thick albumen were extracted for observing the differences in the microwave images. As presented in Figure 10a–d, the microwave image of the fresh egg revealed a weak electrical field because the electromagnetic waves were absorbed by the full albumen. When the thick albumen was extracted, a lower amount of the electromagnetic waves was absorbed by the albumen, and hence, a higher amount of the electromagnetic waves easily penetrated the eggs. The microwave image thus presented a stronger electrical field. Therefore, the proposed waveguide antenna microwave imaging sensing system is useful for quickly identifying the health status of a large number of eggs. The comparison of the performance in the microwave imaging sensing system with previous works is summarized in Table 2.

## 4. Conclusions

In this paper, a waveguide antenna microwave imaging sensing system for food security sensing was demonstrated. The high efficiency transmitting waveguide antenna with 7–13 GHz of scanning frequency and 17.37 dBi of maximum gain was proposed and fabricated. The circular and triangular high-*k* materials were used as testing samples for observing the microwave images in the different dielectric materials. The waveguide antenna microwave imaging sensing system could easily sense the high-*k* materials with different dielectric constants, thicknesses, and shapes, as shown in the produced images. By using the characteristics of the proposed waveguide antenna microwave imaging sensing system, an egg with thin albumen, thick albumen, and yolk can be sensed to show the different electric field distributions on a microwave image, which can be used to evaluate the health of an egg. The waveguide antenna microwave imaging sensing system provides a simple, non-destructive, effective, and rapid method for the application of food security.

## Figures and Tables

**Table 1:** Detailed dielectric constants and conductivity of albumen and yolk.

	Dielectric Constant *ε*_r_ (*f*)			Conductivity (*f*)(S/mm)		
	8.4 GHz	9.2 GHz	10.4 GHz	8.4 GHz	9.2 GHz	10.4 GHz
Albumen	48.0	46.8	45.2	0.0099	0.0112	0.0132
Yolk	28.0	27.4	26.5	0.0055	0.0062	0.0073

**Table 2:** Comparison of other previous works (AS is the area size of scanning, GFS is the ground-folded slot).

Ref.	Antenna Type	*f* (GHz)/Gain (dB)	Object Imaging Type	AS (cm^2^)/Speed (s)	Applications
[5]	Monopole	3–18/4.1	Contour	500/×	Metal
[6]	GFS	1.75–2.1/10.7	Contour	3600/×	Metal
[7]	Aperiodic cavity	18–26.5/×	Contour	2500/×	Metal
[8]	Array Vivaldi	3.1–10.6/6.17	Position	×/×	Medical
[9]	Array slot	0.65–0.96/1.6	Position	×/×	Medical
[10]	Horn	3–11/×	Contour Position	100/×	Medical
This work	Waveguide Horn	7–13/17.37	Contour Position Capacity	900/300	Metal Medical Food
